# 572 Burn Epidemiology in Drug Users

**DOI:** 10.1093/jbcr/irae036.206

**Published:** 2024-04-17

**Authors:** Jaynie X Criscione, Ellie Randolph, Rithvic Jupudi, Sarah Moffitt, Bilal Koussayer, Nicole K Le, Kristen Whalen, Kristina Buller, Jared Troy, Jake Laun

**Affiliations:** University of South Florida, Tampa, FL; University of South Florida, Virginia Beach, VA; USF Health Morsani College of Medicine, Tampa, FL; Morsani College of Medicine USF, Tampa, FL; University of South Florida, Tampa, FL; University of South Florida, Virginia Beach, VA; USF Health Morsani College of Medicine, Tampa, FL; Morsani College of Medicine USF, Tampa, FL; University of South Florida, Tampa, FL; University of South Florida, Virginia Beach, VA; USF Health Morsani College of Medicine, Tampa, FL; Morsani College of Medicine USF, Tampa, FL; University of South Florida, Tampa, FL; University of South Florida, Virginia Beach, VA; USF Health Morsani College of Medicine, Tampa, FL; Morsani College of Medicine USF, Tampa, FL; University of South Florida, Tampa, FL; University of South Florida, Virginia Beach, VA; USF Health Morsani College of Medicine, Tampa, FL; Morsani College of Medicine USF, Tampa, FL; University of South Florida, Tampa, FL; University of South Florida, Virginia Beach, VA; USF Health Morsani College of Medicine, Tampa, FL; Morsani College of Medicine USF, Tampa, FL; University of South Florida, Tampa, FL; University of South Florida, Virginia Beach, VA; USF Health Morsani College of Medicine, Tampa, FL; Morsani College of Medicine USF, Tampa, FL; University of South Florida, Tampa, FL; University of South Florida, Virginia Beach, VA; USF Health Morsani College of Medicine, Tampa, FL; Morsani College of Medicine USF, Tampa, FL; University of South Florida, Tampa, FL; University of South Florida, Virginia Beach, VA; USF Health Morsani College of Medicine, Tampa, FL; Morsani College of Medicine USF, Tampa, FL; University of South Florida, Tampa, FL; University of South Florida, Virginia Beach, VA; USF Health Morsani College of Medicine, Tampa, FL; Morsani College of Medicine USF, Tampa, FL

## Abstract

**Introduction:**

Our state has the second highest rate of drug overdose deaths in the country, with an epicenter located in our greater metropolitan area. Burn patients using drugs can fall victim to burns commonly related to drug use or risky behavior while intoxicated. As our institution is the safety net for this community, we aimed to analyze burn presentation and outcomes of burn patients with active drug use to help clinicians anticipate challenges related to drug usage and limited socioeconomic support.

**Methods:**

Retrospective cohort analysis was performed of patients over the age of 18 years presenting to our institution from 2015 to 2020 with burn injuries. We considered drug use to include marijuana, intravenous drug use, cocaine, methamphetamines, opiates, and alcohol use disorder. We analyzed admission rate with logistic regression, controlling for differences in inhalation burns, related traumatic injury, and other confounding factors.

**Results:**

We reviewed a total of 1339 burn patients with 30.19% reporting drug use. On presentation, burn patients using drugs had a greater percentage of full thickness burns (+0.56%, p< 0.02), TBSA (+1.84%, p< 0.03), burn related trauma (+8.53%, p< 0.01), and concomitant inhalation burns (+3.65%, p< 0.01). Flame burns were most common overall, with scald (-6.15%) and explosion burns (+2.61%) showing the greatest change in frequency for patients using drugs (p< 0.03).

During their hospital course, a larger proportion of burn patients using drugs were admitted (+16.75%, p< 0.01), and drug use was associated with a 2.4 increased log odds of admission (p< 0.01, 95% CI: 1.7-3.4). LOS, surgical intervention, ICU days, and infection showed no significant difference between cohorts. Patients using drugs were less likely to follow-up (-7.39%, p< 0.01) after discharge.

**Conclusions:**

Burn victims who use drugs do not experience clinically significant increase in burn severity, such as TBSA and depth, compared with non-drug users, but were more likely to have inhalation burns and associated traumatic injury. A larger proportion of burn patients using drugs were uninsured or on Medicaid, suggesting the presence of unmanaged chronic conditions that could better explain higher rates of admission after controlling for their burns.

Drug use furthermore where more likely to be lost to follow-up, possibly attributed to decreased access to healthcare secondary to insurance status and limited financial resources. Therefore, we must carefully consider discharge disposition and barriers to care to minimize post burn complications.

**Applicability of Research to Practice:**

We aim to help clinicians treat burn patients who use drugs by anticipating challenges related to their usage and limited socioeconomic support.

